# One size does not fit all: how type of menopause and hormone therapy matters for brain health

**DOI:** 10.1192/bjp.2025.52

**Published:** 2025-06

**Authors:** Laura L. Gravelsins, Liisa A. M. Galea

**Affiliations:** Campbell Family Mental Health Research Institute, Centre for Addiction and Mental Health, Toronto, Ontario, Canada; Djavad Mowafaghian Centre for Brain Health, University of British Columbia, Vancouver, British Columbia, Canada; Department of Psychiatry, University of Toronto, Toronto, Ontario, Canada; Department of Psychology, University of Toronto, Toronto, Ontario, Canada

**Keywords:** Menopausal hormone therapy, vasomotor symptoms, ageing, dementias/neurodegenerative diseases, oestradiol

## Abstract

**Background:**

Menopause is an inflection point in the ageing trajectory. Independent of chronological age, menopause is associated with the biological ageing of several body systems. In this review, we highlight the importance of considering the influence of menopause – its types, symptoms and interventions – on brain health. Supplementing the loss of ovarian hormones with menopausal hormone therapy (MHT) may be key for supporting the healthy brain ageing of females. MHT has been associated with reduced risk of several neurodegenerative diseases; however, its benefits are not always observed on brain health.

**Aims:**

This narrative review highlights often overlooked MHT factors that influence its effects to produce positive or negative effects on brain health, cognition and neurodegenerative disease risk. These factors include the many varieties of MHT, including formulation, administration route and dosing schedule, as well as individual characteristics, particularly the presence of vasomotor symptoms and apolipoprotein ε4 (APOE4) genotype.

**Method:**

PubMed and Scopus were used to identify articles with relevant search terms.

**Results:**

Menopause factors, including age, abruptness and symptoms, influence brain ageing. MHT influences brain health, with transdermal MHT showing more positive effects on brain ageing, but its effectiveness may depend on individual factors such as genotype, reproductive and lifestyle factors.

**Conclusions:**

To develop effective and individualised MHT treatments, further research is needed. Preclinical models must consider the type of human menopause and MHT. To achieve the greatest dementia prevention in females, more menopause education and care is needed that extends beyond 60 years of age, or 10 years postmenopause.

## Menopause is an ageing inflection point

Ageing-related neurodegenerative disorders show sex differences in prevalence.^1^ Females have twice the lifetime risk of Alzheimer’s disease, whereas amyotrophic lateral sclerosis and Parkinson’s disease are more prevalent in males with advancing age. Ageing affects all body systems, with some systems showing prominent sex differences,^2^ including gonadal ageing.^3^ Research suggests that menopause is an ageing inflection point in females that is independent of chronological age. For example, menopause is related to increased epigenetic ageing^4^ and other biological ageing markers that predict mortality.^2,5^ This menopausal inflection point is seen across a wide variety of biological systems and has implications for a wide variety of diseases that manifest with ageing, including cardiovascular disease and stroke, osteoporosis, several autoimmune diseases (such as rheumatoid arthritis, lupus and multiple sclerosis), metabolic disorders (diabetes), cancers and neurodegenerative diseases.^6,7^ These findings suggest that menopause is important to consider in brain ageing research.

**Fig. 1:**
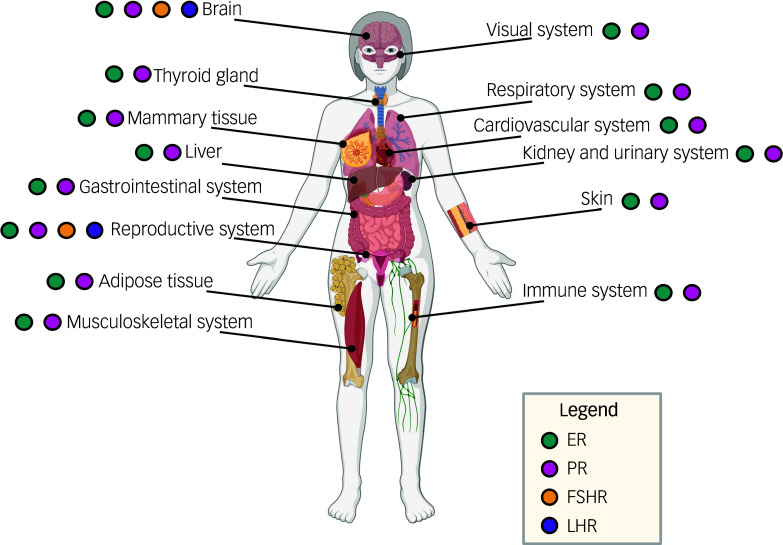
Location of hormone receptors throughout the female body. Oestrogens and progesterone influence all body systems and are densely populated throughout the brain. Follicle stimulating hormone (FSH) and luteinising hormone receptors are also located in the brain. The many menopausal symptoms are not surprising given the widespread distribution of these hormone receptors. The localisation of FSH receptors has not been thoroughly investigated in humans; however, work in rodents suggests that FSH receptors may be located in adipose tissue, bone, heart, kidneys and lungs.^10^ PR, progesterone receptor; ER, estrogen receptor; FSHR, follicle stimulating hormone receptor; LHR, luteinising hormone receptor. Figure created using BioRender.com.

It should, perhaps, not be surprising that menopause is a pivotal point in the ageing process, with widespread influences. Menopause is characterised by a loss of ovarian hormones (oestrogens, progesterone (P4)), and an increase in gonadotropin concentrations (luteinising hormone, follicle stimulating hormone (FSH)). These hormones act on hormone receptors that are located in many parts of the brain and body, and are important for maintaining brain health (see Fig. 1). More work has examined the effects of depletion of oestrogens and P4 on the brain; however, it is important to acknowledge that FSH and luteinising hormone have receptors that are located in the brain as well. Although FSH and luteinising hormone receptors have a lower brain density than receptors for oestrogens and progestogens, some evidence shows that higher levels of FSH and luteinising hormone are associated with worse cognitive outcomes.^8,9^ Thus, menopause-related changes in ovarian hormones and gonadotropins work in concert, and are all likely to contribute to brain health.

Importantly, the profile of gonadal ageing, and respective hormones, substantially differs by sex and gender. ‘Sex’ is defined as biological characteristics that differ between males, females and intersex individuals, whereas ‘gender’ is a psychosocial construst that encompasses not only identity but also society’s expectations of roles and behaviours based on gender identity. In human males, FSH and luteinising hormone play an important role in spermatogenesis and testosterone production, respectively. FSH and luteinising hormone levels increase by approximately 1–2% per year after 40 years of age in males, which causes gradual declines in testosterone levels over time.^11^ In human females, FSH and luteinising hormone play a role in ovulation and oestradiol (E2) and P4 production. FSH and luteinising hormone levels increase by approximately ten-fold,^12^ E2 levels decrease by approximately six- to ten-fold,^12^ and P4 levels decrease by approximately three-fold^13^ over the menopausal transition, which typically begins in the 40s and can last 2–10 years.^14^ Menopause is a significant, female-specific hormonal transition with important implications for ageing. However, fewer than 3% of studies in neuroscience and psychiatry focus on female health,^15^ and fewer than 0.2% of neuroscience studies focus on menopause. These recent statistics highlight that female health research continues to be underfunded and undervalued.

## Overview and methodology

**Fig. 2:**
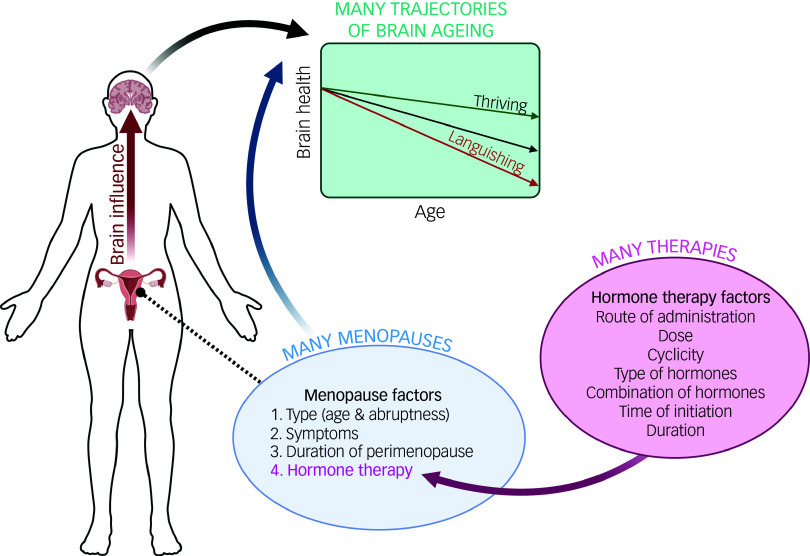
Understanding female brain ageing requires us to do more than investigate sex and gender differences in brain ageing. Female-specific health experiences, like menopause, must be considered in research. Importantly, there is not one type of menopause and there is not one type of menopausal hormone therapy. As depicted, both menopause and hormone therapy can vary in several dimensions. Embracing these complexities in research is necessary to uncover factors that influence individual differences in brain ageing. Figure created using BioRender.com.

In this review, we highlight the importance of considering menopause in brain health research. We provide an overview of the different forms of menopause – spontaneous, early, premature and induced – which differentially affect brain ageing. We discuss the many and highly variable symptoms of menopause and challenges pinpointing the onset of the menopause transition. Moreover, we discuss the effects of menopausal hormone therapy (MHT) on memory and neurodegenerative disease risk, covering often overlooked MHT factors that influence its efficacy and likely contribute to mixed findings. To advance research on MHT’s influence on the brain and cognition, we discuss the pros and cons of rodent models of menopause to maximise their translational value. Finally, we close with recommendations and future research directions to help develop effective, individualised MHT to achieve the greatest dementia prevention in women (Fig. 2).

To identify relevant articles for our narrative review, we searched PubMed and Scopus, using the search terms ‘menopaus, perimenopaus, menopause transition, surgical, age, aging, menopause type, early, premature, hormone therapy, hormone replacement, estrogen, estradiol, progesterone, progestin, testosterone, gonadotropin, luteinising hormone, LH, follicle stimulating hormone, FSH, menopaus symptom, vasomotor, hot flash, night sweat, subjective, cogniti, cognitive decline, brain, memory, rodent, menopause model, ovariectomy, OVX, 4-Vinylcyclohexene diepoxide, VCD, apolipoprotein, APOE4, Alzheimer’s disease, dementia, neurodegener’. Articles published before January 2025 were of interest.

## Menopause types

There are many types of menopause with distinct hormonal profiles and distinct influences on the brain and cognition.

In humans, the ovarian cycle typically ranges from 21 to 35 days, but can vary in length or be absent owing to pregnancy, use of hormonal contraception, stress, exercise, medication or medical conditions that affect ovarian function (e.g. polycystic ovary syndrome (PCOS)^16^). Eventually, ovarian function declines because of the natural depletion of primordial ovarian follicles, resulting in changes in cycle length, and marking the beginning of perimenopause. In some individuals, cycles may initially become more regular before they become more irregular later on in the transition;^17^ this heterogeneity is important to consider when pinpointing the onset of perimenopause.

The perimenopausal stage is characterised by the initiation of menopausal symptoms (further described in section ‘The many menopausal symptoms’), and substantial change in ovarian hormones and gonadotropins. During perimenopause, there is heightened FSH release from the pituitary gland, low levels of anti-Mullerian hormone (a marker of ovarian reserve) and highly variable/fluctuating 17β-oestradiol (E2) release from the ovaries.^7,18,19^ Over time, the ovaries become less responsive to FSH. This results in reduced E2 and luteinising hormone production, more frequent anovulation (i.e. less frequent menses) and, in turn, reduced P4 production until the final menstrual period (i.e. 12 months without menstruation).^7,18,19^ However, as noted below, there is substantial variation in the levels of these hormones, making it challenging to determine thresholds.^12,20^

Although menopause is often referred to as the cessation of ovarian hormones, the postmenopausal ovaries continue to produce testosterone up to 10 years after the final menstrual period.^21^ In addition, there are other sources of oestrogens aside from the ovaries. Adipose tissue, which can increase in postmenopause, can produce another of the oestrogens, oestrone (E1).^19^ Furthermore, the adrenal glands continue to produce androstenedione, a precursor of oestrogens, into older age, but to a much lesser extent than the ovaries.^19^ There is substantial interindividual variability in the concentration of FSH and luteinising hormone as well as in the length of the perimenopausal period, making it challenging to model. On average, perimenopause lasts approximately 4 years, but in some individuals it can be as short as 2 years or upward of 10 years,^14^ with ethnic/geographic disparities^22^ and differences in racial disparities: Black and Hispanic females have been found to experience menopause earlier, on average, than White females.^23^ These intersectional factors are important to consider for understanding the effects of menopause on brain and cognitive outcomes.

Menopause can vary in the age at which it occurs and in the abruptness of its transition, and based on these parameters, can be categorised into spontaneous, early, premature or induced (chemically or surgically). However, the type of menopause is not always accounted for in studies, but can dramatically affect brain health outcomes.^24^ Early and premature menopause follow the same transition and progression of hormonal changes as spontaneous menopause, but occur earlier in time. The definition of early menopause varies, but it is generally defined as menopause before age 45 years and after age 40 years, and premature menopause is defined as menopause before age 40 years. Primary ovarian insufficiency ((POI), also referred to as premature ovarian failure) is distinct from premature menopause. In POI, the ovaries stop functioning optimally before age 40, but individuals with POI can still become pregnant and may experience occasional menstrual periods.^25^ Thus, POI should not be confused with premature menopause.^26^ Both early and premature menopause are experienced by one in ten females.^27^ An earlier age of menopause is linked with poorer cognitive and brain outcomes, as well as increased risk for dementias.^28,29^ Early menopause has also been associated with increased neuropathology, reduced cognition and increases in biological ageing markers. There are region-specific increases in levels of phosphorylated tau, a neuropathological feature of Alzheimer’s disease, in those with high amyloid-β load in the entorhinal cortex in people that have experienced an earlier age of menopause compared with older ages of menopause.^30^ In addition, early age of menopause increases epigenetic ageing, a marker of biological ageing.^4^ Earlier age of menopause is associated with lower verbal memory scores^31^ and lower global cognitive scores in older age, particularly in those with heightened vascular risk factors,^29^ compared with those with later age of menopause.

Menopause can also vary in the abruptness of its transition. Unlike the spontaneous menopausal transition, which typically lasts several years, the menopause transition is abrupt when induced. Menopause can be temporarily induced chemically with gonadotropin-releasing hormone analogues (e.g. leuprolide for endometriosis), but is reversible with the cessation of treatment. Within 2–4 weeks of leuprolide acetate treatment, ovarian hormones are within the postmenopausal range,^32^ whereas gonadotropins significantly decrease. Leuprolide acetate has been associated with worse verbal memory, executive functions and working memory in younger, premenopausal females.^32,33^ In response to chemotherapy and/or radiotherapy, most females will experience a loss of menstrual periods owing to reduced ovarian functioning, and additionally may experience treatment-induced menopause.^34^ The hormonal profile of those with treatment-induced menopause is not well documented and is highly variable depending on the age of the individual, as well as the dose, frequency, duration and type of chemotherapy and/or radiotherapy treatment.^34^ The brain changes and cognitive deficits related to treatment-induced menopause are challenging to disentangle from the neurotoxic effects of the chemotherapy or radiotherapy itself.^35^ Aromatase inhibitors (e.g. letrozole, anastrozole, exemestane) are often administered as adjuvant therapy to those with hormone receptor-positive breast cancers, as they block the conversion of androgens to oestrogens.^36^ Aromatase inhibitors significantly decrease E2 levels, and significantly increase FSH and luteinising hormone levels. Both the human and non-human literature has suggested that aromatase inhibitors may negatively affect brain health and cognition, but the evidence is mixed.^36–39^ Previous tamoxifen use may modulate the influence of aromatase inhibitors on cognition, but this is often overlooked in the human literature.^36,37^

Finally, menopause can be induced surgically by bilateral oophorectomy (i.e. the surgical removal of both ovaries), which results in an immediate and steep decline in E2, P4 and testosterone, and a sharp increase in luteinising hormone and FSH at the time of surgery. Compared with ovarian conservation, individuals with premenopausal bilateral oophorectomy have increased symptoms of anxiety and depression,^40^ greater risk for dementias and accelerated cognitive decline.^41^ Bilateral oophorectomy is a risk-reducing procedure for women with the breast cancer gene (*BRCA*) mutation or other genetic risk factors for ovarian cancer (e.g. *BRIP1*, *RAD51C*/*D*, *PALB2* and *ATM*).^24^ Approximately 300 000 people in the USA experience surgically induced menopause caused by bilateral oophorectomy each year.^42^ Furthermore, bilateral oophorectomy can be accompanied by removal of the uterus (hysterectomy) and/or fallopian tubes (salpingectomy), which can have differing effects on brain health outcomes.^43^ These differing causes of menopause are worth investigating because transition differences and age differences may matter in terms of outcomes on brain health, and yet are not always accounted for in the literature.

### Why is early identification of the menopause transition important?

Ovarian hormones play a role in brain health throughout life. Subjective cognitive complaints are common in perimenopause and reported by one- to two-thirds of females.^44^ Declines in verbal memory are often observed, but objective cognitive changes in other cognitive domains are inconsistently reported during perimenopause.^45^ Evidence suggests that cognitive changes across the menopausal transition depend on a number of factors, including timing and type of menopause. In addition to cognitive changes, brain changes are also observed, further suggesting that the menopausal transition represents an inflection point in the brain ageing trajectory for females.^6^ Independent of chronological age, menopause is related to a shift in metabolism and bioenergetic deficits consistent with an Alzheimer’s disease phenotype,^46^ region-specific changes in grey matter volume^47^ and changes in the underlying neural circuitry that supports episodic memory – one of the earliest cognitive domains affected in Alzheimer’s disease.^48^ Collectively, these findings suggest that menopause may be a critical time for intervention to support healthy brain ageing. Better knowledge of the earliest signs and biomarkers of menopause can help with the early initiation of ageing interventions.

## The many menopausal symptoms

All types of menopause are often accompanied by the presence of menopausal symptoms. The Menopause Rating Scale-II (MRS-II) is a validated questionnaire for assessing the impact of menopausal symptoms on health-related quality of life.^49^ The MRS-II groups 11 menopausal symptoms into three main categories: somatic-vegetative, psychological and urogenital. However, there are many other symptoms that people can experience during and after the menopausal transition than the 11 categorised.^50^ A report from the Menopause Foundation of Canada^51^ lists up to 30 symptoms, with most people reporting seven symptoms at a given time. Vasomotor symptoms ((VMS) hot flashes and night sweats), which can both be present or solely present, are reported with high frequency (around 80%) during the menopausal transition.^52^ Less commonly discussed symptoms of menopause include tinnitus,^53^ burning mouth syndrome (among several other oral health symptoms),^54^ dry eyes, musculoskeletal symptoms (arthritis) and itchiness of the skin, all of which may or may not co-occur with VMS. The sheer variety of symptoms also makes modelling menopause challenging, with many combinations of symptoms possible. Although the variety of symptoms are daunting to study, it should not be surprising considering the widespread abundance of oestrogen and P4 receptors throughout all organs, glands and systems, including the brain. Importantly, these less common symptoms can influence quality of life, but are not captured in questionnaires like the MRS-II.

Even among the more common menopausal symptoms, there is a large variability in their reported prevalence. For example, the prevalence of mood symptoms ranges from 15 to 78% and the prevalence of hot flashes and night sweats ranges from 36 to 87% depending on the study.^55^ This substantial variability is likely attributable to a combination of biological and sociocultural factors^56^ and the severity and type of menopausal symptoms depends on type of menopause. Females with surgically induced menopause experience more severe menopausal symptoms relative to women with spontaneous menopause, and score significantly higher on the MRS-II.^57^ Moreover, severity and type of menopausal symptoms also vary by geographic region. For example, East Asian countries experience fewer and less severe hot flashes compared with European countries.^58^ Another study of Omani females found that they are more likely to self-report physical symptoms compared with cognitive symptoms.^59^ Collectively, these findings suggest that culture may influence openness to endorse/self-report menopausal symptoms and underlying genetic differences may influence the experience of menopausal symptoms. Other studies have also reported that education level, socioeconomic status and working status can influence the severity and type of menopausal symptoms.^60^ Questionnaires like the MRS-II are useful to evaluate the influence of menopausal symptoms on quality of life, but may not accurately capture the menopausal experience of all females, nor, as discussed above, does it capture all symptoms of menopause. This symptom variability makes menopause difficult to model clinically and preclinically, especially given the lack of understanding of how these hormones can influence health in females.

The Stages for Reproductive Aging Workshop (STRAW) criteria – the gold standard for determining menopause – states that using menopausal symptoms to determine menopausal status is not reliable, given their high variability in presentation.^18^ This is an interesting conclusion, given that the STRAW criteria also recommend that FSH levels, in conjunction with frequency of menstrual periods, can be used to determine menopausal status. Although a cut-off of 40 IU/L FSH may be useful for determining menopausal status, repeated measurements of FSH are needed because there is substantial interindividual variability in FSH levels.^20^ Whether a single FSH cut-off point is useful is debatable, given literature showing FSH levels can remain below this cut off point even well into the menopausal transition.^20,45^ Additionally, FSH measurements are not routinely offered in clinical practice for determining menopausal status unless ruling out PCOS, premature menopause, treatment-induced menopause or assessing fertility,^61^ further decreasing their utility. In addition, not including symptoms is limiting menopausal research as phenotypic variability likely has differential needs in terms of treatment and possibly repercussions for brain health, given that VMS are more likely to be tied to white matter hyperintensities.^62^

Another challenge for pinpointing the menopausal transition is that the frequency of menstrual periods is highly variable from person to person. For example, individuals with hysterectomy (i.e. removal of the uterus) have a complete cessation of menstrual periods following surgery, as there is no endometrial lining to be shed each month. Although menopause is known to occur on average 2–3 years earlier in those with hysterectomy compared with those without it,^63^ ovarian function remains normal and cycling occurs, but without menstrual bleeding. Additionally, with hormonal contraception being taken by approximately 400 million individuals worldwide,^64^ it can be increasingly challenging to determine the onset of perimenopause. Depending on the type of hormonal contraception (e.g. hormonal intrauterine device or continuous use of oral contraception without placebo/inactive pills), complete cessation of menstrual periods may be experienced before menopause. Worldwide, 182 million (19%) women aged 15–45 years use long-acting, reversible contraception (2% implant and 17% intrauterine device).^64^ In the USA, use of an intrauterine device increased from 7.1% in 2006–2010 to 21.4% in 2015–2019,^65^ suggesting that it is becoming a more popular choice of contraception. It is also challenging to determine perimenopause onset in women with POI and PCOS, where frequency of menstrual periods is diminished, but not necessarily reflective of ovarian function.

Another challenge is that menopause remains a taboo subject. A recent 2023 report published by the Menopause Foundation of Canada^51^ suggests that menopause is viewed in a negative light, and initiating conversations related to menopause is a challenge; 50% of females worry that menopausal symptoms will affect how they are perceived at work, and 30% believe that if they disclosed their menopausal symptoms then others will perceive them as weak, old or ‘past their prime’. In addition, people report dissatisfaction in their menopausal care. Informed menopause care from health care providers is often challenging, given the lack of knowledge and awareness of the variety of menopausal symptoms (see section ‘Recommendations and future directions’).

## What is MHT?

MHT is used to supplement the decline in ovarian hormones during the menopausal transition, to offset symptoms. For females with an intact uterus, concurrent progestin/P4 is needed to prevent endometrial hyperplasia and cancer.^66^ Although serum testosterone levels remain relatively stable throughout perimenopause and several years postmenopause, concurrent testosterone may be recommended as part of the MHT regimen, especially when lower sexual drive, without an underlying cause, is observed.^67^ Testosterone may also be offered as part of the MHT regimen to females with POI or ovarian removal, who may have reduced or no testosterone output from the ovaries. Females with premenopausal oophorectomy have 25% lower circulating testosterone compared with spontaneously menopausal females with intact ovaries.^68^ It is important to note that many types of MHT are available, with varying doses and combinations of oestrogens, progestins and possibly androgens.

MHT can vary in several dimensions that affects its benefits – not only for alleviating menopausal symptoms, but also for ensuring healthy brain ageing into later life. These dimensions include its formulation, dose, route of administration (transdermal, oral, vaginal, intramuscular), cyclicity (continuous versus cyclic), time of initiation and duration. Additionally, individual health histories and biologies need to be considered in assessing which type of MHT may be the best, including the health status of the person receiving MHT, pregnancy history, genetic predispositions for dementia, as well as their age and type of menopause (healthy cell bias hypothesis, timing hypothesis). In the following sections, we will review the evidence pertaining to these factors, highlighting gaps in our knowledge and important research areas to consider moving forward.

To date, unfortunately, one of the most influential MHT studies is the Women’s Health Initiative Study (WHIS), a large randomised clinical trial that administered continuous oral conjugated equine oestrogen (CEE) and medroxyprogesterone acetate (MPA), or continuous oral CEE-alone therapy (to those with a hysterectomy), or placebo to females aged 50–79 years.^69^ Initially, they reported increased risk for breast cancer, dementia, stroke and pulmonary embolisms after combined treatment.^70,71^ These findings resulted in the premature discontinuation of the WHIS and massive changes in MHT prescribing practices. Prescriptions for combined CEE and MPA decreased by 66% in the USA from January to June 2003 relative to January to June 2002.^72^ The estimated number of MHT users was approximately 35 million before the WHIS, halved in the early 2000s and stabilised to 12 million MHT users in the 2010s.^73^ However, the study design has been widely criticised on a number of factors.

The WHIS results pertain to one type, dose, dosing schedule and route of administration of MHT, and one type of menopause. In addition, the WHIS had few exclusions for participants, as it included individuals at high risk for cardiovascular disease and approximately a third of participants were obese or smoked. Also, people with more severe VMS, who may be most likely to benefit from MHT (see section ‘VMS and brain health’), were discouraged from participating in the trial.^69^ Follow-up studies stratifying by age and time since menopause observed that increased risk of heart disease was only observed in those ages 60 years and beyond 10 years since menopause.^74^ Additionally, an evaluation of younger women who were aged 50–55 years at the start of the trial showed that taking CEE was not linked to adverse cognitive outcomes.^75^ Moreover, brain atrophy and dementia risk was greatest in those with worse baseline cognitive scores, as measured by the Mini-Mental State Examination,^76,77^ suggesting that the benefits from MHT may be limited to prevention of cognitive decline rather than the treatment of cognitive decline.

These findings are in line with the ‘window of opportunity hypothesis’ or ‘timing hypothesis’, which posits that initiating hormone therapy close to menopause onset has cognitive benefits, whereas initiating it further from menopause onset may have no effects or produce cognitive decrements. Meta-analyses support the initiation of hormone therapy during the early menopausal period.^78,79^ Indeed, studies show that the brain’s responsiveness to exogenous hormones declines after an extensive period of ovarian hormone deprivation. Consistent with this, in rodents a greater time since ovariectomy was associated with reduced neural stem cells within the dentate gyrus.^80^ Moreover, in humans, oestrogen receptor expression in the brain may change over the course of reproductive senescence, as postmenopausal females have greater oestrogen receptor density in certain regions compared with premenopausal females;^81^ however, using ^18^F-fluoroestradiol positron emission tomography imaging for *in vivo* detection of brain oestrogen receptors has been challenged.^82^ The potential upregulation of brain oestrogen receptors in postmenopause may reflect a compensation for reduced circulating levels of oestrogens. However, the Kronos Early Estrogen Prevention Study (KEEPS) did not find that early MHT initiation positively influenced cognition.^83^ Importantly, the transdermal E2-treated group had more baseline white matter hyperintensities and a larger proportion of *APOE4* carriers than the placebo group in the KEEPS trial,^84^ which may have masked the cognitive benefits of E2. Additionally, MHT may have cognitive benefits in older age, depending on the type of hormones, route of administration and cyclicity/dosing schedule.^85,86^ MHT has been postulated to be preventative not curative, such that MHT may be beneficial only in healthy individuals, and more likely be detrimental in unhealthy individuals. According to the ‘healthy cell bias of oestrogen action’, uncompromised neurons respond positively to exogenous hormones.^87^ This may explain why MHT has been associated with brain and cognitive benefits in older individuals, but is more likely to be beneficial if initiated closer to the onset of menopause.

MHT formulations containing E2 are more likely associated with beneficial effects compared with formulations containing E1, perhaps because of the more potent effects of E2 on ERs. In fact, E2 binds with nearly two-thirds as much strength as E1 to oestrogen receptors.^88^ Interestingly, E1 is the main component of CEE, which was administered in the WHIS. Other studies have shown greater verbal memory benefits with E2 over CEE in younger postmenopausal females^89^ (controlled for menopause type), as well as decreased rates of ventricular expansion and white matter hyperintensity volume compared with placebo in younger, spontaneously menopausal females.^84^ Work in ovariectomised female rodents, a surgical menopause model, has demonstrated that E2 increases, whereas E1 decreases, hippocampal neurogenesis,^80,90^ and E2 facilitates, whereas premarin (E1) impairs, working memory.^91–93^ Collectively, these findings suggest E2 may be the more beneficial MHT type to promote brain plasticity and memory.

**Fig. 3:**
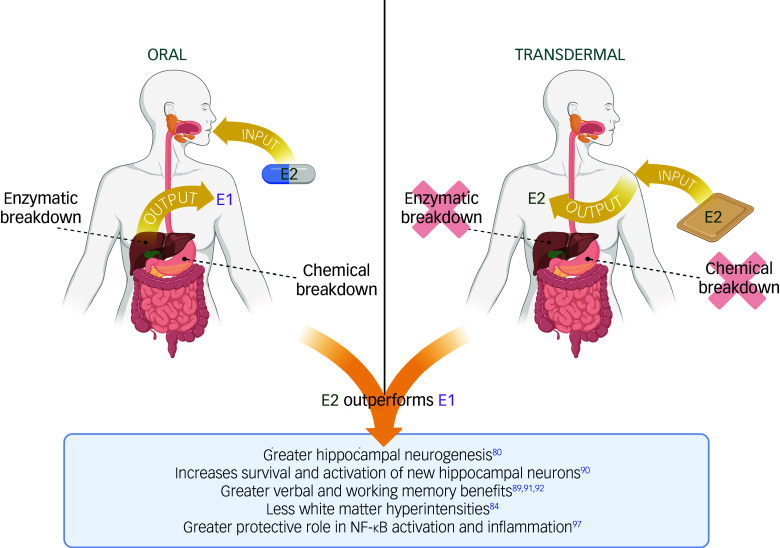
Oral versus transdermal routes of administration of oestradiol-based menopausal hormone therapy. When taken orally, oestradiol is subject to a large first-pass metabolism effect; it undergoes chemical breakdown by the gut as well as enzymatic breakdown by the liver, and is largely converted to oestrone. Transdermal routes of administration largely bypass first-pass metabolism, resulting in more bioavailable oestradiol. Several lines of research suggest that oestradiol outperforms oestrone in its brain and cognitive benefits.^80, 84, 89–91, 97^ E1, oestrone; E2, oestradiol; NF-κB, nuclear factor-kappa B. Figure created using BioRender.com.

Although E2-based MHT may be more beneficial, it is important to consider that when it is taken orally, it is largely converted to E1 because of a high first-pass metabolism effect, meaning it is broken down by the liver and gut (Fig. 3). Both transdermal and vaginal routes of administration of E2 bypass first-pass metabolism, resulting in more constant hormone levels, more bioavailable E2 and an E1:E2 ratio that more closely approximates premenopausal levels compared with oral oestrogens.^88^ Moreover, transdermal and vaginal oestrogens pose a lower risk for venous thromboembolism, as they result in lower clotting factors and have a more favourable risk profile of markers of cardiovascular heart disease than oral oestrogens.^94^ Female rodent studies of CEE have shown that its negative effects on cognition are dose-dependent, only occurring with high CEE doses.^92,95^ There is a strong biological rationale that transdermal and vaginal HT preparations likely have brain and cognitive benefits over oral preparations. One study found oral preparations reduced risk for several neurodegenerative diseases,^96^ but type of hormones was not considered in this analysis. It remains unclear whether transdermal preparations of E2 specifically provide superior protection against neurodegenerative diseases than oral preparations.

Similar to the type of oestrogens, the type of progestogen treatment may influence cognition. P4 is associated with greater brain and cognitive benefits than MPA, a progestin (i.e. synthetic P4). Unlike P4, MPA is associated with reduced antioxidant defences within the hippocampus,^98^ and fails to increase brain-derived neurotrophic factor (BDNF) within the cortex.^99^ Moreover, unlike P4, MPA has been linked to worse spatial reference memory and worse spatial working memory in ovariectomised female rats.^100^ Furthermore, MPA co-administration blocks E2’s neuroprotective effects in the hippocampus and dentate gyrus.^101,102^ MPA is also associated with a higher risk of venous thromboembolism compared with P4.^103^ These findings stress that not all MHT regimens are equal in terms of their cognitive and brain benefits.

Nearly two decades after the WHIS, its study findings continue to influence perception of MHT risks, warning labels displayed on MHT monographs and MHT prescribing practices. One lasting negative outcome of the WHIS trial includes amplified worry surrounding increased risk of breast cancer, stroke and dementia with MHT use. In fact, even warning labels on low-dose vaginal oestrogen products (e.g. 0.01 mg doses of Vagifem) list increased risk of stroke, blood clots and dementia as potential risks associated with their use (https://pdf.hres.ca/dpd_pm/00073342.PDF). However, there has been pushback from medical professionals to remove these warnings^104^ in light of scientific evidence to suggest the contrary. Vaginal oestrogens result in significantly lower systemic oestrogens compared with oral oestrogens. Moreover, a large cohort study of women found no increased risk of breast cancer-specific mortality with the use of vaginal oestrogens following breast cancer diagnosis, even in women with past oestrogen receptor-positive breast cancers.^105^ Similarly, another large cohort study found no increased risk of breast cancer with the use of low-dose transdermal oestrogens,^106^ as well as several benefits associated with taking oestrogens alone beyond 65 years of age. These benefits included reductions in dementia, mortality, lung cancer, colorectal cancer, congestive heart failure, venous thromboembolism, acute myocardial infarction and recurrent urinary tract infections.^104,106^ These studies suggest that lower-dose oestrogens with vaginal or transdermal routes of administration have several benefits, even in older menopausal females. Thus, the benefits of MHT must be appropriately balanced with the risks for the given individual.

## VMS and brain health

As discussed, there are many symptoms of menopause, all of which can influence quality of life and potentially influence cognition and the brain. VMS, night sweats and hot flashes, are the most studied, which are hypothesised to arise from declining ovarian hormone levels, particularly oestrogens, that drive changes in neurotransmitter levels and instability in the hypothalamic thermoregulatory center.^55^ Hot flashes are distinct from night sweats, but they often co-occur.^107^ The prevalence of VMS increases over the menopausal transition, with as many as 80% of postmenopausal women reporting these symptoms at a moderate to severe level.^108^ Most females self-report four to five hot flashes per day, but this can be as high as 20 per day.^109^ The number of objective night-time hot flashes is approximately 3.5, and approximately 90% are accompanied by night sweats.^107,110^ VMS can contribute to sleep disruption.^110^ VMS can be measured subjectively by asking participants to self-report how many hot flashes and night sweats they experienced, as well as measured objectively/physiologically via sternal skin conductance. Although there is good correspondence between these two methods of quantifying VMS, females generally underreport (rather than overreport) the number of physiologically measured hot flashes experienced.^109^ VMS are associated with reduced quality of life, but also measurable cognitive and brain changes.

Physiologically measured VMS have been associated with worse verbal memory, independent of sleep, age, ethnicity and mood, and some evidence suggests that treating VMS can improve memory performance.^111^ Beyond cognition, VMS have also been associated with biomarker and brain changes.^62,112^ In ovary-intact females not taking MHT, more severe and later-occurring self-reported VMS (i.e. VMS that appeared further from menopause onset) were associated with accelerated epigenetic ageing – a marker associated with greater physical ageing and premature death – compared with females without VMS.^113^ Additionally, later-occurring self-reported VMS were also associated with an increased risk of cardiovascular disease and all-cause mortality.^114^ Interestingly, if VMS appeared closer to the onset of menopause, these associations did not persist. Thus, there are many temporal trajectories of VMS onset, as well as varying degrees of VMS severity, that influence cognition, Alzheimer’s disease brain biomarkers and cardiovascular health.

Additionally, experiencing more VMS has been linked to greater white matter hyperintensity burden in women currently not taking MHT. More frequent physiologically recorded hot flashes were associated with greater whole brain white matter hyperintensities that were not explained by cardiovascular disease risk factors, depressive symptoms, endogenous E2 or sleep fragmentation.^62^ A subregion analysis showed that VMS were most strongly associated with white matter hyperintensity volume in the frontal lobe. On the contrary, self-reported hot flashes were not associated with white matter hyperintensity volume.^62^ Together, these findings suggest that the frontal lobe may be most vulnerable to VMS-induced brain changes and physiologically recorded VMS may be a more sensitive predictor of brain changes. Although MHT is considered the most effective first-line treatment for alleviating VMS by the Society for Obstetricians and Gynecologists of Canada, the American College of Obstetricians and Gynecologists and the Canadian Cancer Society of Canada, there is insufficient evidence as to whether MHT is helpful for preventing white matter hyperintensity burden. One recent study found that any MHT use beyond 5 years postmenopause was associated with greater white matter hyperintensity burden;^115^ however, this seemingly contradictory linkage of MHT to greater white matter hyperintensity burden may be related to the later initiation of MHT and lack of consideration of MHT types. More objectively measured VMS have also been associated with greater brain amyloid-β pathology in women not taking MHT,^112^ suggesting that more VMS may increase Alzheimer’s disease pathology.

In some females, persistent hot flashes are experienced beyond 60 years of age; one study found that 10% of females continue to experience hot flashes in their 60s and 5% in their 70s.^116^ This suggests that VMS can persist beyond the recommended time of MHT cessation, which is age 60 years or >10 years postmenopause. Given the association of VMS with brain changes, this guideline should be reconsidered. Importantly, VMS also cause sleep disruption, and greater sleep disruption has been associated with increased white matter hyperintensities independent of VMS.^117^ It is possible that VMS may negatively influence cognition and the brain through their effects on sleep.^111^ Sleep problems are common in the menopause transition, and great care should be taken to treat these sleep problems given their strong associations with neurodegenerative disease.^55^ Furthermore, the presence of severe menopausal symptoms was related to an increased odds for developing mild cognitive impairment (MCI), a prodromal state to Alzheimer’s disease.^118^ Females with MCI experience more severe menopausal symptoms across all three subscales (somatic-vegetative, psychological and urogenital symptoms) of the MRS-II than females without MCI (mixture of menopause types),^118^ independent of age, years of education and body mass index. Importantly, this suggests that menopausal symptoms beyond hot flashes and night sweats should be investigated for their potential role in cognitive and brain changes and treated aggressively. Knowing which menopausal symptoms might be driving increased risk for MCI is key for developing effective therapeutics.

## Apolipoprotein 4 genotype

Apolipoprotein E (APOE) plays a role in fat metabolism and cholesterol transport. Carriers of the *APOE4* variant, which is associated with disrupted cholesterol homeostasis,^119^ are at a significantly greater risk for sporadic (i.e. non-familial) Alzheimer’s disease. Homozygous *APOE4* carriers show as high as a 15-fold increased risk for Alzheimer’s disease compared with non-carriers, with heterozygous and homozygous female carriers at even greater risk than male carriers.^120^ In addition, female carriers show more Alzheimer’s disease neuropathology (i.e. greater cerebrospinal fluid amyloid-β, greater total tau and elevated tau-to-amyloid-β ratio) compared with male carriers.^121^ Thus, *APOE4* status is a non-modifiable risk factor for Alzheimer’s disease that confers a greater risk in women.

Interestingly, *APOE4* status may influence MHT’s effect on the brain and cognition. Some findings suggest that female *APOE4* carriers may benefit more from MHT than non-carriers. Current or past MHT (oestrogens alone or combined with progestogens) was associated with greater entorhinal and amygdala volumes, as well as higher delayed memory score, in *APOE4* carriers only.^122^ An earlier age of MHT initiation was also associated with larger hippocampal volumes in *APOE4* carriers only, suggesting that they may be more responsive to MHT.^122^ Indeed, MHT initiation before menopause is associated with less brain ageing, but only in *APOE4* carriers.^123^ Moreover, a recent prospective clinical study showed that *APOE4* carriers had significantly lower pathophysiological Alzheimer’s disease plasma biomarkers if they took MHT, compared with those who did not.^124^ Together, these studies suggest that MHT may have more brain benefits for *APOE4* carriers.

Despite these findings, other studies have found the opposite. *APOE4* carriers with past or current MHT showed faster cognitive decline than those who never used MHT.^125^ Furthermore, a few rodent studies suggest that E2 has more benefits for neuroprotection following ovariectomy in non-carriers as compared with *APOE4* carriers.^126,127^ It remains to be determined why these differential effects are seen. One potential explanation is that these studies in rodents were done in young animals, suggesting perhaps a biphasic MHT effect in *APOE4* carriers; oestrogens may be detrimental earlier in life, but beneficial later in life. In fact, the effects of the *APOE4* allele itself on cognition vary over time. Carrying *APOE4* is associated with cognitive benefits in midlife, particularly for executive functions, but with cognitive risks at age 65 years and beyond.^128^ Given that *APOE4*’s effects on cognition may vary over time, it is not surprising that MHT may have differential brain effects in *APOE4* carriers over time. Moreover, menopause type has not been investigated. Most rodent studies of menopause use the ovariectomy model of menopause, a form of induced menopause that has more severe cognitive consequences than natural/spontaneous menopause. Also, one study found that only carriers with two *APOE4* alleles – but not one *APOE4* allele – had negative responses to MHT, suggesting that the number of *APOE4* alleles may influence MHT effects.^127^ Future work that considers age, menopause type and number of *APOE4* alleles – and their interactions – is needed to further our understanding of MHT’s effects on the brain and cognition.

## Modelling human menopause in rodents

Having appropriate, well-designed models of menopause in non-human animals is necessary for furthering research on MHT and developing tailored MHT treatments that translate to the best ageing outcomes in females. In the following section, we discuss rodent models of human menopause. In rodents, natural ageing, ovariectomy and chemical induction of menopause are used to model different types of human menopause. The model chosen has an influence on outcomes, much like the differential effects of menopause type on MHT effects or disease risk (Table 1). Although it is beyond the scope of this review, non-human primate models of menopause are also serve as valuable means to further our knowledge on MHT and female brain ageing.^129,130^ We urge the research community to consider both the menopause model and type of menopause in humans to further scientific discovery.

**Table 1:** Summary of rodent menopause models and their important considerations

Human menopause type	Rodent model	Rodent model considerations
Surgically induced	Ovariectomy	Surgery timing – does the age of ovariectomy align with the age of human surgically induced menopause? Hormone therapy – is the type, route of administration, dose and scheduling similar to the menopausal hormone therapy used in humans?
Spontaneous menopause	Natural ageing	Daily lavage – is daily lavage being conducted to accurately determine the trajectory of reproductive senescence of the animals? Mixed groups – similar to humans, there is not one type/trajectory of reproductive senescence
Chemically induced	VCD	Timing of VCD injections – are injections being administered to postpubescent animals? Weight loss – are injections being administered using an established protocol to minimise weight loss? Toxic/teratogenic – is an appropriate dose being used to lessen the toxic, non-ovarian effects of VCD?

VCD, 4-Vinylcyclohexene diepoxide.

### Comparing rodent reproductive senescence to human menopause

Similar to humans, rodents experience reproductive senescence and accompanying hormonal changes. Middle-aged mice and rats (approximately 9–12 months old, timing may differ by species and strain) experience a significant increase in FSH over the course of reproductive senescence, as well as irregular cycles, irregular ovulation and decreased fertility.^131^ The rodent equivalent of human postmenopause, persistent dioestrus, typically occurs at approximately 14–18 months old, and is characterised by low E2 and P4, and elevated luteinising hormone and FSH.^132^ Much like the human menopause, the timing of the rodent persistent dioestrus depends on genotype and environment.^133^ However, before transitioning from irregular cycles to persistent dioestrus, at approximately 12 months old about 60–70% of rodents enter constant oestrus (also referred to as persistent oestrus).^131^ In constant oestrous, there are moderate to high levels of E2 and P4, and a lack of ovulation and preovulatory luteinising hormone surge.^131,134^ The rodent can also directly transition from irregular cycles to persistent dioestrus, which occurs approximately 25–40% of the time.^131,134^ Thus, there are many possible trajectories from irregular cycles to persistent dioestrus. This parallels the human menopause transition, which varies substantially in length and levels of ovarian hormones from person to person. Thus, even in aged rodents, daily lavage is necessary for at least 8 days to determine the type of transition to persistent dioestrus.

### 4-Vinylcyclohexene diepoxide as a model for induced menopause

To model the ovarian follicle depletion in rodents, 4-Vinylcyclohexene diepoxide (VCD) may be used, a chemical agent that targets ovarian follicles leading to a model of induced menopause.^56^ Repeated dosing of VCD results in the gradual depletion of primary and primordial ovarian follicles, but the retention of residual ovarian tissue. The mechanism of VCD-induced follicular loss is similar to the apoptotic mechanism of reproductive senescence-induced follicular loss in humans.^135^ The ovaries of VCD-treated rodents continue to secrete androgens,^136^ which is also similar to the ovaries of postmenopausal humans. An advantage of using the VCD rodent model of induced menopause is that the dosing schedule can be carefully controlled to manipulate the length of the perimenopausal period and the age at menopause onset, two factors that are highly variable in the human population.^14,22,23^

However, VCD is a carcinogen that can have negative effects on non-ovarian tissue. Studies suggest that VCD can cause damage to the stomach, kidneys and liver in a dose-dependent manner.^131,137^ Another important point to consider is the age of initiation of VCD treatment; earlier VCD studies used young prepubertal rodents.^136,138^ When using VCD to model induced menopause, it is important to consider the developmental stage of the rodent to more accurately model the human menopausal transition. VCD introduces a menopause phenotype in middle-aged female rats 11–12 months old,^139^ in which complete follicular depletion occurred 99 days after first VCD injection, intermittent oestrus and dioestrus stages, and undetectable or low levels of serum E2 and P4, elevated luteinising hormone and FSH, and unaltered androstenedione.^139^ More VCD studies in middle-aged rodents that document ovarian hormone and gonadotropin changes before menopause are needed for modelling induced menopause.

### Ovariectomy as a model of surgical menopause

Ovariectomy, surgical removal of the ovaries, models human surgically induced menopause. Similar to humans, within hours of ovariectomy, there is a steep increase in FSH^140^ and steep decline in oestrogens and P4, such that they are undetectable in serum within days post-ovariectomy.^141^ This hormonal profile is more consistent with human surgically induced menopause than human spontaneous menopause. Moreover, the cognitive changes associated with ovariectomy, including spatial working memory and reference memory decrements,^142,143^ overlap with those observed in human surgically induced menopause.^144,145^ These consistencies reinforce the utility of the ovariectomy model as a model of surgically induced menopause.

The ovariectomy model has given important insight into the molecular mechanisms underlying brain and cognitive changes following human surgically induced menopause. In particular, the ovariectomy model has provided important biological support for the critical window/timing hypothesis of MHT initiation. Over time post-ovariectomy, activity of BDNF decreases,^146^ the number of neural stem cells within the dentate gyrus decreases,^80^ and oestrogen receptor degradation increases, particularly within the hippocampus.^147^ These findings point to reduced capacity of hippocampal neurogenesis following ovarian removal, which may be related to reduced BDNF activity and loss of oestrogen receptors. In fact, similar to human studies, earlier initiation of MHT post-ovariectomy has been associated with more favourable cognitive and brain outcomes.^142,148^ Together, these findings suggest that the ovariectomy model is useful for uncovering mechanistic factors that drive MHT response.

Attention is also needed when investigating MHT effects in rodents. Given that the route of administration can influence the type and levels of circulating oestrogens (covered in ‘What is MHT?’), it should be considered in rodent studies investigating MHT effects on the brain and cognition. It is worth noting that rodent subcutaneous routes of administration are more similar to human transdermal routes of administration, as they produce stable serum concentrations of hormones^149^ and bypass first-pass metabolism. Although microneedle transdermal MHT patches have been developed for use in hairless rodents, they cause discomfort and may introduce other confounds (e.g. rodents eating surgical tape used to adhere the patches^150^). Oral oestrogens have been used, and rodent vaginal routes of administration exist, but are less widely used as they require surgical implantation and may require additional surgeries depending on the required length of treatment.^151^ Being mindful of the same MHT factors – like route of administration, type of hormones and dosing schedule – in rodents is important to further scientific discovery and ensure good correspondence with human findings.

## Recommendations and future directions

There is not one type of perimenopause, menopause or postmenopause. Similarly, there is not one type of MHT. Given this, it is unrealistic to expect that one type of MHT will equally benefit all females, who have different genetics and varied life experiences. Females and healthcare practitioners alike must appreciate these nuances to ensure all females receive informed, individualised menopausal care to support their healthy brain ageing.

This starts with improved menopausal education. Approximately 65% of females do not feel prepared for menopause,^152^ and most general practitioners acknowledge that they are lacking menopause education, especially education surrounding menopause management and various forms of MHT.^153^ One survey found that only 6.8% of USA medical practitioners felt sufficiently prepared to support females through the menopause transition.^154^ In fact, only 31.3% of USA obstetrics and gynaecology residency programmes reported having a menopause curriculum.^155^ This is concerning, knowing that 38% of females feel that their menopausal symptoms are undertreated,^51^ and in Canada alone, 540 000 days of work are lost each year because of ineffective menopausal symptom management.^51^ Unmanaged menopausal symptoms negatively influence quality of life and have negative economic consequences. This lack of menopause education increases the possibility that menopausal symptoms, especially if they deviate from the commonly reported VMS, may be left unrecognised or mistakenly attributed to other causes. In current medical practice, the onus falls on the patients to determine when their menopause transition is commencing and to advocate for treatments to help ease this transition. There are significant delays in diagnosis across disorders in females compared with males, and females feel their symptoms are dismissed in healthcare practitioner offices.^156^ Coupled with the findings that healthcare practitioners are not equipped with knowledge on menopause, it is imperative that more research and education of healthcare practitioners takes place. Given that 50% of the population will go through menopause and that just less than half of the female lifespan will be spent in a perimenopausal or postmenopausal state, it is shocking so little attention has been paid to menopause care.

Compounded bioidentical hormone therapy (cBHT) has exploded in popularity, partly because of the lack of appropriate care by healthcare practitioners and physician prescriptions are not needed to obtain it. cBHT is taken by up to 40% of females in the USA.^157^ cBHT is marketed as a more ‘natural’ option; however, it undergoes chemical extraction and stabilisation processes.^158^ It is overlooked is that many prescribed MHTs also use natural hormones of oestradiol, E1 or even oestriol and P4. Moreover, cBHT is not approved by Health Canada, meaning that their safety and efficacy have not been evaluated with the same standards as prescribed MHT. In fact, there is no procedure to ensure cBHT contains the listed amount of active ingredient, and many cBHT formulations use E1.^158^ The safety and efficacy of prescribed MHT is influenced by the type, route of administration and dose of oestrogens and progestogens. These same factors influence the safety and efficacy of cBHT, but they have not been formally regulated. Current guidelines suggest that more research is needed on the efficacy of cBHT, and it is recommended that their use be restricted to patients with a known allergy to an active pharmaceutical agent.^158^

### When is it appropriate to stop MHT?

Most guidelines recommend the cessation of MHT at 60 years of age or 10 years after menopause, guidelines that are still informed by the WHIS findings.^159^ Additionally, because of the WHIS findings, many physicians believe that MHT duration should be limited to 5 years or less,^160^ despite the average duration of VMS being approximately 7 years.^108^ Notably, a significant proportion of females – nearly 40%^161^ – experience the resurgence of menopausal symptoms following the discontinuation of MHT, even if the MHT dose is gradually tapered. In light of research linking more severe menopausal symptoms to MCI,^118^ research associating VMS with brain and sleep disturbances and research indicating minimal or decreased cancer risk with MHT (especially with non-oral routes of administration), whether there should be a push to stop MHT after a certain age or time of use is debatable. This is especially true for those with persistent menopausal symptoms (e.g. females who continue to experience hot flashes into their 70s) and in the case of surgically induced menopause caused by bilateral oophorectomy, where there is a complete absence of postmenopausal ovarian hormone production.

In transgender and gender-diverse populations, gender-affirming hormone therapy continues into older ages. Risks of continuing care are balanced with the need to reduce gender dysphoria and feminisation/masculinisation effects.^162^ The risks for continuing MHT care in cisgender females beyond age 60 years or beyond 10 years after menopause should be balanced with the need to reduce menopausal symptoms and improve quality of life. Other MHT options should be considered rather than strict adherence to an age or duration cut-off; lower, effective MHT doses with transdermal or vaginal routes of administration should be offered.^105,106^ The potential of brain-selective oestrogens for maintaining brain health should also be explored. The prodrug 10β,17β-dihydroxyestra-1,4-dien-3-one (DHED) can be systemically administered, but converts to E2 only in the brain. DHED has shown promise in preserving working and recognition memory in ovariectomised mice, and providing neuroprotection in rat stroke models and improving sleep in disturbances in ovariectomised marmosets.^163–165^ The risk and benefits of MHT need to be updated and appropriately balanced with the emergence of new scientific evidence.

### Avenues for future research

In addition to considering genetic risk factors for Alzheimer’s disease, like *APOE4* status, in evaluating MHT’s influence on cognition and the brain, other reproductive and lifestyle factors need to be considered. The menopausal period is often studied in isolation, but there are several other reproductive health transition periods that may also interact with MHT status. Pregnancy complications (e.g. preeclampsia) may set a trajectory for poorer brain health.^166^ Parity (number of children) history influences *APOE4*’s effects on the brain and cognition,^167^ and use of hormonal contraception has been associated with reduced cognitive impairment and reduced dementia risk.^28^ Although pregnancy and hormonal contraception use may occur decades before menopause, the substantial ovarian hormone changes during these periods may result in lasting brain changes that influence an individual’s response to MHT (see Box 1 below). Additionally, several other modifiable dementia risk factors, including obesity, hypertension, diabetes, physical activity, smoking, cognitive engagement and social interaction,^168^ should be addressed to optimise brain health. Importantly, MHT can interact with these risk factors, which likely works to influence dementia and Alzheimer’s disease risk.^169^ Moreover, stress and trauma influence menopausal symptoms,^170^ further suggesting that life experience could have important implications for reproductive senescence and MHT response. In addition to taking a ‘lifespan approach’ to studying menopause, there is a pressing need to further our understanding of the connection of menopausal symptoms to brain health, given their associations with MCI and dementia brain pathology.^112,117^ Menopausal symptoms are often studied in isolation (e.g. only investigating VMS), but females experience an average of seven symptoms at a given time.^51^ Moreover, the nature and severity of menopausal symptoms depends on culture and menopause type;^56,57,118^ however, Western populations with spontaneous menopause are largely studied. Considering the collective impact of menopausal symptoms on brain health, within the context of cultural diversity and differing menopause types, should be a priority in developing targeted menopause treatments. Embracing these complexities in research is a necessary path forward to develop tailored MHT treatments with the greatest brain benefits for females.

Box 1 Key research questions

Do other menopausal symptoms, aside from VMS, drive cognitive and brain changes in females? How does this differ by intersectional factors such as ethnicity and economic status?

Do other female-specific experiences, including hormonal contraception, menstrual cycle characteristics, parity and length of the perimenopausal period, influence MHT’s effects on the brain and cognition?

Does MHT route of administration influence the brain and cognition?

What combination of treatments for menopausal symptoms (e.g. MHT, cognitive–behavioural therapy, exercise, diet change) is most effective for symptom alleviation and does this differ for different symptom constellations?

What brain and cognitive benefits, if any, does MHT provide for older females with persistent menopausal symptoms (i.e. symptoms that continue beyond age 60 years and beyond 10 years since menopause)?

Menopause is a pivotal inflection point in the ageing trajectory. Early identification of the menopausal transition and furthering our MHT knowledge is key for initiating appropriate care to preserve brain health in females. This starts with better-quality female health research that considers the many varieties of MHT and menopause. Several lines of research point to transdermal oestradiol as an effective form of MHT with brain and cognitive benefits. However, its effectiveness may depend on individual factors, such as APOE genotype, reproductive history and life experiences. Embracing this complexity in research is necessary to identify effective, individualised MHT. Rodent models present an attractive option to further our knowledge of the biological and environmental factors that affect an individual’s MHT response; they provide mechanistic insights into the actions of MHT on the brain and cognition. Importantly, the appropriate model of rodent menopause must be carefully selected to most accurately model the type of human menopause and MHT in question. In addition to dedicated research, more menopause education is needed. Healthcare practitioners need to proactively initiate conversations about menopause with their female patients. The undertreatment of menopausal symptoms suggests that more menopause specialists are needed. Specialists must be well-versed and kept up to date with the many types of menopause, the many symptoms of menopause and the many varieties of MHT including their risks and benefits. To support healthy brain ageing, menopause care beyond 60 years of age or 10 years postmenopause is needed. Half of the human population will go through menopause, yet our knowledge about menopause and brain health is considerably lacking. We must prioritise research that embraces the many menopause types, symptoms and therapies, and support all females in receiving informed and continued menopause care into later life.

## Data Availability

Data availability is not applicable to this article as no new data were created or analysed in this study.
